# The neurobiology of human sequential signal prediction: Insights from language, music, and mathematics

**DOI:** 10.1016/j.neures.2025.105001

**Published:** 2026-01

**Authors:** Tomoya Nakai, Tatsuya Daikoku, Yohei Oseki

**Affiliations:** a Araya Inc., Tokyo, Japan; b LaPsyDÉ, CNRS UMR8240, Université Paris Cité, Paris 75005, France; c Graduate School of Information Science and Technology, University of Tokyo, Tokyo, Japan; d Centre for Neuroscience in Education, University of Cambridge, Cambridge, United Kingdom; e Graduate School of Arts and Sciences, University of Tokyo, Tokyo, Japan

**Keywords:** Language, Music, Mathematics, Statistical learning, Hierarchical structure

## Abstract

Humans use various sequential signals, such as language, music, and mathematics, to convey complex information and facilitate communication. Previous research has identified two fundamental frameworks underlying human sequential signal processing: a structural framework, emphasizing rule-based hierarchical organization (e.g., syntax), and a predictive framework, which focuses on the brain’s capacity to anticipate upcoming inputs based on statistical regularities. While the structural approach underscores human-specific abilities, the predictive approach highlights mechanisms shared with non-human animals. We review behavioral and neural evidence across domains, demonstrating overlapping neural substrates, particularly in the inferior frontal gyrus, involved in structural processing of language, music, and mathematics. Likewise, predictive processing, indexed by ERP components such as N400 and P600, operates across domains to detect violations of expectation. Importantly, we argue that these frameworks are not mutually exclusive: structural knowledge can inform prediction, and predictive processes can, in turn, influence perceived structure. Cross-domain experiments and computational modeling suggest shared cognitive mechanisms, although domain-specific variations remain. We propose that the human brain integrates hierarchical structures with statistical learning to support flexible and generalized sequence processing. Future research should aim to develop unified models across domains, leveraging neuroimaging techniques and large language models.

## Highlights

• Humans process diverse sequential signals, including language, music, and mathematics.
• Sequential signals share both structural and predictive properties across domains.
• Predictive mechanisms include components like N400 that reflect expectation violations.
• Structural mechanisms involve the inferior frontal gyrus across domains.
• Human sequence processing integrates hierarchical structures with statistical learning.

## 1 Introduction

Humans use various types of sequential signals to express information and communicate with one another, including language, music, and mathematics. Sequential signals convey information not only through the elements themselves, but also through the order in which they appear. Such signals are typically time series, but can also include those obtained from serial gaze scanning. These signals are segmented into discrete chunks, and by assigning specific symbols to each chunk, humans can encode them as symbolic sequences. Sequential signals and their symbolic representations are pervasive throughout human societies. Language, for instance, plays a central role in human communication (Holler and Levinson, 2019, Tomasello, 2010). Music constitutes a significant component of cultural identity (Jacoby et al., 2024, Mehr et al., 2019). Most modern technologies would not function without mathematical calculations (Crosby, 1997, Hansson, 2020). Although communication based on sequential signals has been observed in other animals (Abe and Watanabe, 2011, Arnon et al., 2025, Lameira et al., 2023, Sainburg et al., 2019), human sequential signals can span multiple cognitive domains and serve as the foundation of complex culture and technology.

**Fig. 1:**
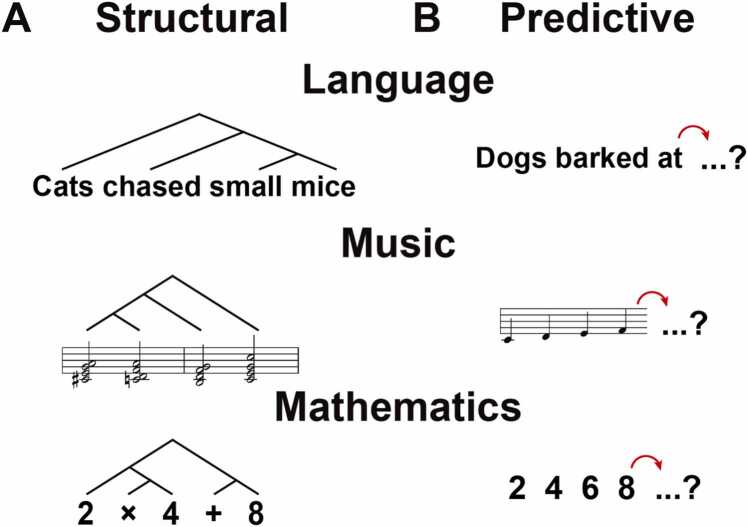
Structural and predictive aspects across human sequential signals. (A) Structural aspect: Human sequential signals have hierarchical structures. In language, syntactic structures determine sentence meaning (e.g., “Cats chased small mice”). In music, harmonic progressions (e.g., A7–Dm7–G7–C) create a strong resolution. In mathematics, mathematical expressions consist of combinations of numbers and operators (e.g., 2 × 4 + 8). (**B**) Predictive aspect: Human sequential signals are processed in a predictive manner. In language, words are predicted based on context. In music, listeners expect the next chord or note in a progression. In mathematics, mathematical expressions lead to the prediction of the result. These shared aspects suggest both structural and predictive mechanisms.

Previous studies have emphasized two fundamental properties that characterize human sequential signals across domains. The first is the *structural* aspect (Fig. 1**A**). Sequential signals convey different meanings not only through individual elements (chunks) but also through the way these elements are arranged. For example, in language, the word sequences “Cats chased small mice” and “Mice chased small cats” convey distinct meanings, despite consisting of the same lexical elements. In music, the II–V–I chord progression (e.g., Dm7–G7–C), a canonical sequence in jazz and Western tonal music, follows a subdominant–dominant–tonic structure that establishes a strong sense of resolution. In contrast, the I–II–V progression, though not harmonically incorrect, begins on the tonic and lacks a clear sense of closure. In mathematics, the numerical expressions “2 + 4 × 8” and “2 × 4 + 8” contain the same elements but yield different computational outcomes. These examples illustrate how the ordering of identical elements in language, music, and mathematics alters functional interpretation and perceived coherence. Note that in this review, the term “sequential” refers solely to the properties of the signals themselves, and does not imply that human processing mechanisms operate in a sequential manner (as assumed in, for example, Markov models). To account for these phenomena, particularly in language, theoretical linguistics has introduced a set of explicit rules, namely syntax, that govern the ordering of elements within sequential signals (Chomsky, 2008, Chomsky, 1995). Within this rule-based framework, researchers have proposed that humans possess a unique capacity for processing sequential signals (Berwick and Chomsky, 2017, Fujita and Fujita, 2021, Hauser et al., 2002). According to this view, a set of operations, such as Merge, generates hierarchical structures and may have evolved as neural mechanisms unique to the human brain.

In contrast, the *predictive* aspect (Fig. 1**B**) reflects the fact that sequential signals are inherently temporal and that humans process them according to the order of input. For example, when reading a sentence such as “Dogs barked at …”, we naturally expect the next word to be something like “strangers”, “cats”, or “boys”, entities that dogs typically bark at, rather than unrelated words like “idea” or “banana”. Similar predictive mechanisms can also be observed in music and mathematics. When listening to a melody such as “do, re, mi, fa…”, we naturally anticipate the next note to be “so”. When presented with a numerical sequence like “2, 4, 6, 8…”, we confidently predict that the next number will be “10”. Even when multiple signals are presented simultaneously on a screen, humans tend to scan them sequentially with their eyes and predict the most likely subsequent element (Demberg and Keller, 2008, McDonald and Shillcock, 2003). Importantly, the ability to predict sequential signals is not unique to humans. Prediction plays an essential role in the dynamic processing of sequential signals in the brains of many species (Santolin and Saffran, 2018), including songbirds (Lu and Vicario, 2014, Ono et al., 2016), dogs (Boros et al., 2021), and monkeys (Kikuchi et al., 2017). Therefore, the framework of predictive processing offers a valuable perspective for understanding human cognition in continuity with that of other animals.

These two aspects of sequential signals, along with their corresponding research frameworks, offer contrasting perspectives on the differences between humans and other animals. The structural framework tends to emphasize the ways in which humans differ from other animals (Berwick and Chomsky, 2017, Fujita and Fujita, 2021, Hauser et al., 2002). In contrast, the predictive framework highlights the mechanisms shared by humans and other animals (Rey et al., 2019, Sainburg et al., 2019, Santolin and Saffran, 2018). In addition, these two frameworks differ in the temporal scales of information processing they emphasize. The structural framework focuses not on local transitions between elements but rather on their global relationships, i.e., how those elements are combined. In most cases, such structures are described in relation to an entire sequence and tend to ignore the dynamic processing mechanisms across individual elements. In contrast, the predictive framework focuses on the local transition probabilities between elements. It is effective for explaining processing at finer temporal scales but tends to overlook the global relationships among elements. However, these two aspects are not necessarily mutually exclusive; rather, they represent complementary dimensions of human sequential signal processing. Nevertheless, previous studies have tended to emphasize one aspect over the other and an integrative understanding across the two frameworks has been limited.

The present review aims to bridge the gap between these two frameworks of sequential signal processing and their associated neural bases. We propose that the human brain possesses shared structural and predictive mechanisms for processing a variety of sequential signals, with a particular focus on language, music, and mathematics. These mechanisms are particularly suited for processing hierarchically structured sequences, making human sequence processing distinct from that of other animals. In the following sections, we first outline the theoretical background of the structural and predictive frameworks of sequential signal processing. We then present behavioral and neural evidence from studies on language, music, and mathematics, focusing on both structural and predictive aspects. Finally, we propose a perspective that integrates both structural and predictive aspects of sequential signals.

## 2 Theoretical background

In this section, we provide the theoretical background for both structural and predictive frameworks and discuss how they function in sequential signal processing. The structural framework can be broadly divided into two categories: one that formally defines sequential signals, and another that analyzes their structures based on actual linguistic data. The former is exemplified by automata theory, whereas the latter is primarily employed in theoretical linguistics. The predictive framework has been widely adopted in neuroscience through the theory of predictive coding, which is not limited to sequential signals and has also contributed to the development of statistical learning in human language processing. More recently, its integration with information theory has led to the development of quantitative metrics, such as the concept of surprisal.

### 2.1 Structural framework

#### 2.1.1 Automata theory

The formal analysis of sequential signals is captured by automata theory (Chomsky, 1956, Hopcroft et al., 2006), which classifies sequential signals of varying complexity based on the computational capacity of different automata (see Hopcroft et al. 2006 for the formal definitions of automata). Different classes of formal grammars that can generate sequential signals, along with their corresponding automata, are organized within the Chomsky hierarchy. Each type of automaton is capable of recognizing only specific patterns due to its architectural limitations. For example, finite automata can recognize simple repetition patterns such as ABABAB, namely patterns of the form (AB)^n^ (for positive integers *n*), but they cannot recognize patterns such as AAABBB, namely patterns of the form A^n^B^n^ where the number of repeats for the first and second symbols is identical. In contrast, pushdown automata can recognize both ABABAB and AAABBB, indicating that the set of symbol sequences recognizable by pushdown automata is a proper superset of those recognized by finite automata. Similarly, patterns of the form A^n^B^n^C^n^ cannot be recognized by pushdown automata but can be recognized by linear bounded automata. The sets of rules that generate sequences recognizable by finite, pushdown, and linear bounded automata are referred to as regular, context-free, and context-sensitive grammars, respectively. Finally, sequences generated by unrestricted grammars, which encompass the most powerful class in the Chomsky hierarchy, are recognized by Turing machines.

Within this hierarchy, human language has been argued to exhibit a mildly context-sensitive grammar (Heinz and Idsardi, 2013). Specifically, certain human languages, such as a dialect of Swiss German, contain constructions that cannot be generated by context-free grammars or recognized by pushdown automata (Shieber, 1985). Automata-based descriptions have also been applied to sequential signals beyond language. Mathematical expressions are typical examples that conform to context-free grammars and are modeled by pushdown automata (Hopcroft et al., 2006). Tonal sequences in music have also been described using context-free grammars (Rohrmeier, 2011). These observations suggest that human sequential signals exhibit at least the complexity associated with context-free grammars.

#### 2.1.2 Hierarchical structures and long distance dependency

In theoretical linguistics, sequential signals, chunked and labeled with symbols, are typically represented as hierarchical syntactic structures. In particular, the Minimalist Program (Chomsky, 2008, Chomsky, 1995), a modern formulation of Chomskyan syntax, proposes that the Merge operation, which simply combines two input elements X and Y to produce an unordered set Z = {X, Y}, forms the core computational mechanism of human language. The output set Z can serve as a new input for the recursive Merge operation; applying Merge to Z and another element W yields {W, Z} = {W, {X, Y}}. Such recursive operations generate hierarchical syntactic structures, often represented using labeled brackets or equivalent tree diagrams. Although the Minimalist Program was developed for analyzing natural language, the abstract Merge operation is, by definition, not limited to the linguistic domain. In fact, theoretical linguists argue that recursive computation in natural language also underlies the natural number system (Chomsky, 2008, Hauser et al., 2002), aligning with the concept of a universal generative faculty shared across language, music, mathematics, and other cognitive domains (Fujita and Fujita, 2021, Hauser and Watumull, 2017, Lerdahl and Jackendoff, 1983, Matsumoto and Nakai, 2023).

Several quantitative measures have been proposed to investigate the behavioral and neural correlates of structural processing (Uddén et al., 2020). For example, the hierarchical level can be defined as the number of recursive embeddings from the root to the deepest terminal node in a given tree diagram (Nakai and Sakai, 2014, Ohta et al., 2013, Pallier et al., 2011). The hierarchical level does not merely reflect the number of Merge operations; it also considers the depth of their recursive application. Other researchers have used distance-based metrics to quantify long-distance dependencies between elements, another major structural feature of natural language (Chen et al., 2022, Levelti, 1970, Liu et al., 2017). For example, in the sentence “What did the teacher say that the student forgot to bring?”, the word *What* functions as the object of the verb *bring*, even though it appears at the beginning of the sentence, far from its canonical position. Distance can be measured as either the number of intervening terminal nodes between two elements (sequential distance), or the number of intervening non-terminal nodes along the path between two elements (hierarchical distance).

### 2.2 Predictive framework

#### 2.2.1 Predictive coding and statistical learning

Predictive coding is a leading theory within the predictive framework (Clark, 2013, Friston, 2005, Friston and Kiebel, 2009, Gabhart et al., 2025, Hohwy, 2013, Rao and Ballard, 1999, Spratling, 2017). According to this view, the brain operates as a predictive system, continuously generating expectations about incoming sensory inputs based on accumulated prior experience. Discrepancies between predicted and incoming sensory inputs, termed prediction errors, are iteratively minimized by refining internal models. This process follows a Bayesian framework, in which prior beliefs about the causes of sensory signals are combined with the likelihood of the observed input to yield plausible posterior beliefs (Friston, 2005, Friston and Kiebel, 2009). Note that, in this review, “predictive processing” denotes a broad collection of theoretical frameworks (adopted not only in neuroscience but also in cognitive science and psycholinguistics) for explaining human sequential signal prediction, while “predictive coding” is a specific algorithmic/neural circuit hypothesis within the framework that posits reciprocal cortical pathways implementing top-down predictions and bottom-up prediction errors with precision weighting (Clark, 2013, Friston and Kiebel, 2009, Hohwy, 2013, Spratling, 2017). In what follows, we use the term “predictive processing” for the overarching view and “predictive coding” when referring to circuit-level implementations.

Building on predictive coding theory, statistical learning serves as a core cognitive mechanism by which the brain constructs and refines internal models to predict sequential signals (Frost et al., 2019, Hasson, 2017). Unlike rule-based systems that require explicit instruction, statistical learning allows the brain to implicitly extract statistical regularities from sequential input and dynamically adjust predictions in response to environmental variability. Initially studied in the context of language (Saffran et al., 1996), where it supports speech segmentation, statistical learning has since been demonstrated across multiple domains, including music (Daikoku, 2018, Daikoku et al., 2021, Pearce, 2005) and vision (Fiser and Aslin, 2001, Fiser and Lengyel, 2022, Schapiro et al., 2012). Notably, statistical learning enables even newborns and infants to detect and segment frequently co-occurring sound patterns, such as words or phrases in speech, despite having no prior linguistic exposure (Fló et al., 2024, Teinonen et al., 2009). At its core, statistical learning involves tracking transitional probabilities between elements in a sequential signal and forming internal models that encode these probabilistic patterns over time. Importantly, statistical learning operates at multiple levels: it encodes local dependencies, such as the likelihood of one element following another, as well as perceptual uncertainty at higher levels of the processing hierarchy (Daikoku, 2023, Okano et al., 2021), thereby modulating the strength of predictions and guiding attentional and neural responses in dynamic contexts.

#### 2.2.2 Information-theoretic complexity metrics

Independently of predictive coding theory, information-theoretic complexity metrics (such as *surprisal* and *entropy*) are commonly employed in psycholinguistics and language neuroscience to quantify predictive processing (Hale, 2016, Hale, 2006, Hale, 2001, Levy, 2008). Surprisal, also known as Shannon self-information (Shannon, 1948), is defined as the negative logarithm of the probability of a word *w*, as follows:

(1) $$I\left(w\right)={-\log}_{2}p(w)$$

Within the predictive framework, it is assumed that humans are constantly anticipating the next word in a sequence. When an incoming word deviates from these expectations, the probability decreases, resulting in a higher surprisal value. For example, given the context “The cat is on the,” the word *mat* is more probable and less surprising than *rocket* as the next word. Entropy, defined as the weighted sum of surprisal, is another metric used to quantify uncertainty over possible upcoming words:

(2) $$H\left(W\right)=-\underset{w\in W}{\sum }p(w)\log_{2}p(w)$$

where *W* is the set of all possible next-word candidates. In human language processing, a decrease in entropy, known as entropy reduction, has been associated with processing effort, as it reflects the extent to which uncertainty is resolved when a new word is encountered (Hale, 2016, Hale, 2006).

An important advantage of information-theoretic complexity metrics is that they can serve as indicators of alignment between computational models and human behavioral or neural data. In particular, with the advent of large language models (LLMs) in recent years, these metrics make it possible to assess the extent to which such models resemble human sequential signal processing (Brennan and Hale, 2019, Goldstein et al., 2022, Heilbron et al., 2022, Schmitt et al., 2021, Tuckute et al., 2024). Moreover, complexity metrics can be applied in naturalistic paradigms, such as listening to extended narratives, which have attracted considerable attention in human neuroimaging research (Hasson et al., 2020, Sonkusare et al., 2019). Such paradigms enable the study of language processing under ecologically valid conditions, providing insights that are more representative of real-world cognition than those obtained from conventional experiments using artificial stimuli.

## 3 Sequential signal processing in language

### 3.1 Structural processing in language

The brain mechanisms underlying structural processing in language have been investigated using non-invasive neuroimaging techniques. Early studies reported activation in the left inferior frontal gyrus (IFG) during the processing of syntactically complex sentences in comprehension tasks (Embick et al., 2000, Musso et al., 2003, Stromswold et al., 1996), suggesting a functional distinction from semantic processing (Dapretto and Bookheimer, 1999, Friederici et al., 2000). In particular, the left IFG responds to sentences composed of pseudowords (i.e., jabberwocky sentences) (Indefrey et al., 2001, Pallier et al., 2011), suggesting that this region is involved in processing abstract hierarchical structures. To further assess structural complexity, sequential signals can be classified according to automata theory, distinguishing between grammars of different computational complexities. Studies using sequential signals derived from artificial grammars demonstrated greater activation in the left IFG for sequences generated by context-free grammars compared to regular grammars (Bahlmann et al., 2008, Friederici et al., 2006). A meta-analysis of similar studies showed that the left IFG and the left posterior superior temporal sulcus (pSTS) are involved in constructing hierarchically structured sentences (Zaccarella et al., 2017).

Subsequent studies have evaluated the processing load of hierarchical syntactic structures using quantitative metrics, enabling more direct analysis of structure-dependent brain activity. For example, Pallier et al. (2011) presented word sequences with increasing constituent sizes and found that activations in the left IFG and left pSTS were modulated accordingly. Similar findings have been reported by modeling brain activity with hierarchical levels of tree diagrams (Ohta et al., 2013, Umejima et al., 2023), dependency distance (Santi et al., 2015), and the maximum number of dependencies (Giglio et al., 2024, Uddén et al., 2022). Using electrocorticography (ECoG), Nelson et al. (2017) revealed dynamic changes in neural activity in the IFG and pSTS associated with the construction of hierarchical syntactic structures. They further found that this activity was modulated by the number of open nodes maintained in working memory. Collectively, these results have paved the way for investigating structure-dependent neural responses in a quantitative manner.

### 3.2 Predictive processing in language

Research on predictive processing includes studies on both semantic and syntactic deviations. In the domain of semantic prediction, Kutas and Hillyard (1980) found that semantically anomalous words in sentences (e.g., “He spread the warm bread with socks”) elicited a large negative brain response, peaking around 400 ms (i.e., N400) after the deviant word. The N400 has also been observed in response to other meaningful stimuli, including faces and pictures (Kutas and Federmeier, 2011). Its amplitude partially reflects stimulus expectancy (Kutas and Hillyard, 1984), and this relationship has been formalized within the predictive framework (Michaelov et al., 2024). In the domain of syntactic prediction, syntactic deviation is associated with two distinct ERP components: early left anterior negativity (ELAN), occurring between 120 and 200 ms (Neville et al., 1991) and the P600, a positive waveform peaking around 600 ms (Osterhout and Holcomb, 1992). These two components are thought to play distinct roles: ELAN is associated with the automatic identification of a word’s syntactic category, whereas P600 reflects sentence-level integration of syntactic and semantic information (Friederici, 2011).

The application of information theory and artificial neural networks has enabled quantitative evaluation of predictive processing in both behavior and brain activity. Studies have used surprisal values to explain reading times (Shain et al., 2024), eye movements (Salicchi et al., 2023), and brain responses (Heilbron et al., 2022). Some studies have utilized artificial neural networks that explicitly incorporate architectures for processing hierarchical syntactic structures. For example, Hale et al. (2018) and Brennan et al. (2020) showed that surprisal calculated using Recurrent Neural Network Grammars (Dyer et al., 2016) can explain EEG and fMRI activity, respectively. Other researchers have calculated surprisal using LLMs to investigate alignment between brain activity and these models (Goldstein et al., 2022, Heilbron et al., 2022). Building upon this line of research, Caucheteux et al. (2023) investigated predictive processing by measuring brain–LLM alignment using future word representations. However, these studies did not find localized prediction in the left IFG or pSTS; rather, brain activity was distributed across the fronto-temporal cortices. This might be due to the fact that most of these studies employed naturalistic paradigms without controlled stimuli.

## 4 Sequential signal processing in music

### 4.1 Structural processing in music

Researchers have attempted to analyze hierarchical structures in music by applying syntactic frameworks developed for natural language (Lerdahl and Jackendoff, 1983, Rohrmeier, 2011, Steedman, 1984). Building on these theoretical frameworks, neuroimaging studies have reported neural responses that reflect the structural properties of musical stimuli. Many of these studies examine brain responses by introducing harmonically irregular chords within otherwise regular chord sequences. A pioneering study using EEG and magnetoencephalography (MEG) demonstrated the early right anterior negativity (ERAN) in response to a harmonically inappropriate “Neapolitan sixth” chord that did not fit the surrounding chord sequence (Koelsch et al., 2000, Maess et al., 2001) and further reported bilateral IFG involvement based on source localization (Maess et al., 2001). Subsequent fMRI studies have reported consistent findings, showing activation in the right IFG when participants were presented with chord sequences containing irregular chords (Bianco et al., 2016, Koelsch et al., 2005, Koelsch et al., 2002, Tillmann et al., 2003). Furthermore, Koelsch et al. (2013) directly modified musical stimuli based on syntactic theory, constructing hierarchical structures according to the context-free grammar proposed by Rohrmeier (2011), and reported ERP responses to irregular chords embedded in these structures, even when local sequential relationships remained unchanged. Using a similar approach with tone sequences, Cheung et al. (2018) found right IFG activation in response to tones that violated hierarchical structures.

Beyond harmonic structures, other studies have focused on rhythmic structures, which involve hierarchical groupings based on beats and meters (Asano and Boeckx, 2015, Fitch, 2011, Heard and Lee, 2020). For example, tapping polyrhythmic (i.e., structurally complex) sequences activates language-related brain regions, including the ventral portion of the bilateral IFG (area 47) (Vuust et al., 2011, Vuust et al., 2006). Using positron emission tomography, Thaut et al. (2014) reported activation in the right IFG and precentral gyrus specifically in response to meter information, but not to other rhythmic features. Furthermore, a meta-analysis by Heard and Lee (2020) demonstrated overlapping activation across studies of musical rhythm and language syntax in the left IFG, left supplementary motor area, and bilateral insula, although rhythm perception additionally engages regions in the right hemisphere. These findings suggest partially shared processing mechanisms for harmonic and rhythmic syntax.

### 4.2 Predictive processing in music

Music serves as a valuable domain for investigating predictive processing in the brain (Koelsch et al., 2019), particularly through its reliance on implicit statistical learning mechanisms. Initially identified as a core mechanism underlying early language acquisition in neonates (Saffran et al., 1996), statistical learning has since been recognized as a crucial process in musical learning, with extensive research supporting its role in shaping auditory perception and cognitive processing (Pearce, 2005). Neural evidence has shown that human pitch prediction in novel melodies is closely aligned with statistical models of transitional probabilities derived from large musical corpora (Pearce et al., 2010, Pearce and Wiggins, 2012, Pearce and Wiggins, 2006), suggesting that the brain encodes internal probabilistic representations of musical structure through statistical learning.

For instance, when individuals are exposed to novel tone sequences containing embedded statistical regularities, early ERP components such as mismatch negativity (MMN) or ERAN are elicited in response to statistically unexpected (i.e., low-probability) transitions (Daikoku et al., 2015, Koelsch et al., 2000). This indicates that the auditory system automatically detects violations of predicted patterns, even without explicit attention. Furthermore, later components such as P300 and N400 have been associated with violations of more abstract or hierarchically structured expectations, reflecting higher-level cognitive engagement with probabilistic structures (Abla et al., 2008, Daikoku, 2018, Koelsch et al., 2016). In addition, recent neuroimaging studies have shown that musical pleasure, as represented in the amygdala and hippocampus, is jointly modulated by uncertainty and surprisal (Cheung et al., 2019). Consistently, Daikoku et al. (2024) demonstrated that musical uncertainty and surprisal elicit distinct bodily sensations associated with emotional valence. These findings imply that the probabilistic features of music not only guide prediction but also shape affective responses.

## 5 Sequential signal processing in mathematics

### 5.1 Structural processing in mathematics

The syntactic structure of mathematical expressions, which can be formally captured by a context-free grammar, follows the same generative rules as those in language (Matsumoto and Nakai, 2023), and people visually scan mathematical expressions according to these structures (Jansen et al., 2007, Schneider et al., 2012). fMRI studies have further shown that language-related brain regions, particularly the left IFG, are involved in processing hierarchical structures in mathematics (Makuuchi et al., 2012, Nakai and Okanoya, 2020, Nakai and Okanoya, 2018, Nakai and Sakai, 2014). Activity in the left IFG varies according to the syntactic complexity of mathematical expressions (Makuuchi et al., 2012, Nakai and Sakai, 2014). Moreover, presenting language and mathematical stimuli in succession results in cross-domain structural priming (Scheepers et al., 2019, Scheepers et al., 2011, Scheepers and Sturt, 2014, Van de Cavey and Hartsuiker, 2016) and adaptation effects in the bilateral IFG (Nakai and Okanoya, 2018), suggesting a shared neural basis for syntactic processing in language and mathematics.

Syntactic structures are also evident in the construction of multi-digit numbers (Hurford, 2007, Hurford, 1975, McCloskey et al., 1986). For example, three-digit numbers such as “123” and “321” convey different quantities, even though they are composed of the same digits. This difference arises from the place-value rule (e.g., 123 = 1 × 100 + 2 × 10 + 3) and the underlying syntactic structure. A behavioral study showed that participants tended to produce number words with syntactic structures similar to those they had previously heard, suggesting a syntactic priming effect for multi-digit number words (Dotan et al., 2021). Neuroimaging studies have implicated the left IFG and the anterior temporal lobe in the syntactic and compositional processing of multi-digit numbers (Blanco-Elorrieta and Pylkkänen, 2016, Hung et al., 2015). These findings suggest that similar syntactic processing mechanisms are involved in constructing both mathematical expressions and multi-digit number units.

### 5.2 Predictive processing in mathematics

One of the most well-known phenomena in the predictive processing of mathematical expressions is the arithmetic N400 (Dickson et al., 2018, Galfano et al., 2009, Niedeggen et al., 1999, Niedeggen and Rösler, 1999). Extending the original concept of the N400, which was first reported in response to semantic anomalies in language (Kutas and Hillyard, 1980), Niedeggen and Rösler (1999) found that when participants were presented with the incorrect equation “4 × 7 = 26,” a negative ERP component emerged between 300 and 500 ms, compared to the correct equation “4 × 7 = 28.” This pattern resembled the semantic N400, supporting the view that humans retrieve multiplication facts from long-term memory in a manner similar to semantic associations in language (Ashcraft, 1992, Chen and Campbell, 2018, Logan, 1988). The brain’s response to arithmetic facts appears to be automatic and unconscious, as it can be detected even when participants are asleep (Strauss and Dehaene, 2018). In contrast, the N400 was not observed in children with dyscalculia, suggesting that this component reflects individual differences in mathematical ability (Cárdenas et al., 2021).

Statistical learning in number series has been investigated through the lens of numerical inductive reasoning (Lang and Kotchoubey, 2002, Liang et al., 2016, Qin et al., 2009, Xiao et al., 2022), in which participants are exposed to number sequences (e.g., 2, 4, 6, …) and implicitly learn hidden rules (e.g., +2 increments). Even when participants are unaware of the regularities in number sequences, their pupil size responds to changes in frequency (Binda et al., 2025). Violations of number sequences elicit different ERP components compared to violations of calculation results. Numerous studies have reported an early negative component (N200) and a late positive component (P600) in response to number sequence violations (Lang and Kotchoubey, 2002, Núñez-Peña and Escera, 2007, Núñez-Peña and Honrubia-Serrano, 2004, Qin et al., 2009), paralleling the P600 effect observed in response to syntactic violations in mathematical expressions (Martín-Loeches et al., 2006). Finally, Jia et al. (2011) and Liang et al., 2016, Liang et al., 2014 demonstrated the involvement of the left IFG and the bilateral parietal cortex in numerical inductive reasoning, consistent with their roles in syntactic processing of mathematical expressions.

## 6 General discussion

In this review, we have argued that there are two research frameworks for analyzing human sequential signals, including language, music, and mathematics. The first framework focuses on structural processing and highlights the role of hierarchical syntactic structures. The second framework relates to predictive processing and assumes that humans and other animals share similar mechanisms. In this section, we propose possible directions for integrating these frameworks.

### 6.1 Shared neural mechanisms across different sequential signals

Previous studies using fMRI and EEG/MEG source localization have reported that sequential signal processing occurs in common brain regions across language, music, and mathematics. Numerous studies have reported the involvement of the IFG in syntactic processing across language (Ohta et al., 2013, Pallier et al., 2011), music (Cheung et al., 2018, Maess et al., 2001), and mathematics (Hung et al., 2015, Nakai and Sakai, 2014). Noteworthy examples include studies that examined cognitive tasks across multiple domains, such as music and language (Asano et al., 2021, Chiang et al., 2018, Kunert et al., 2015), or mathematics and language (Nakai and Okanoya, 2020, Nakai and Okanoya, 2018). By measuring brain activity across multiple cognitive domains within the same group of participants, these studies controlled for the potential influence of group differences. Furthermore, in examining shared neural bases, studies using priming and repetition suppression effects provide more informative insights than those relying solely on overlapping brain activity. This is because association areas such as the IFG are involved in various cognitive functions, making it possible for the same region to be coincidentally activated during unrelated tasks. For example, Nakai and Okanoya (2018) found a cross-domain repetition suppression effect between mathematical expressions and sentences in the bilateral IFG, suggesting that these two cognitive domains interact through shared neural circuits. To the best of our knowledge, Van de Cavey and Hartsuiker (2016) conducted the only study that reported a behavioral priming effect across language, music, and mathematics within the same experiment. Although neural correlates were not examined, this study suggests that common processing mechanisms may exist for a variety of sequential signals.

On the other hand, several studies have reported that the syntactic structure of music is processed in the right IFG (Bianco et al., 2016, Cheung et al., 2018, Koelsch et al., 2005, Koelsch et al., 2002, Tillmann et al., 2003), which is contralateral to that of language. Furthermore, even within the left IFG, syntactic processing of language is associated with the anterior part, including Brodmann areas 45 and 44 (Friederici et al., 2017), while syntactic processing of mathematics tends to be associated with activity in more posterior regions, including area 44 and the precentral gyrus (Makuuchi et al., 2012, Nakai and Okanoya, 2020, Nakai and Okanoya, 2018). In addition, several studies have reported that language and mathematics (Amalric and Dehaene, 2016, Monti et al., 2012, Varley et al., 2005), as well as music and speech (Albouy et al., 2020, Chen et al., 2023, Norman-Haignere et al., 2015), are associated with distinct brain regions. These results suggest the possibility that, even if similar structural processing mechanisms are involved in language, music, and mathematics, the specific brain regions supporting these mechanisms may differ.

In terms of electrophysiological responses, numerous studies have reported the N400 component in response to semantic violations in language (Kutas and Hillyard, 1980), music (Daltrozzo and Schön, 2009, Koelsch et al., 2004, Miranda and Ullman, 2007), and mathematics (Niedeggen et al., 1999, Niedeggen and Rösler, 1999). In particular, Calma-Roddin and Drury (2020) investigated the N400 semantic effect for both language and music using song stimuli with lyrics, and found an interference effect between the language and music N400 responses, suggesting shared neural resources across domains. Regarding syntactic violations, two other ERP components, i.e., P600 and ELAN/ERAN, have frequently been reported. The P600 has consistently been observed in response to syntactic anomalies in language (Hagoort et al., 1993, Osterhout and Holcomb, 1992), music (Patel et al., 1998, Sun et al., 2018), and mathematics (Martín-Loeches et al., 2006, Núñez-Peña and Honrubia-Serrano, 2004). In contrast, a notable difference among the three domains is the involvement of the ERAN in music, which has been found on the cortical side contralateral to the ELAN in language (Friederici et al., 1996), possibly reflecting different functional roles for the P600 and ELAN. In sum, these findings suggest that EEG/MEG responses reflect similar predictive patterns across language, music, and mathematics, based on anticipation of upcoming signals.

So far, we have focused on previous research in language, music, and mathematics, but studies have also investigated the brain mechanisms underlying sequential signal processing in other domains. In particular, studies that present shapes in a sequential manner have the potential to bridge geometric pattern perception with other human sequential signals (Dehaene et al., 2025, Dehaene et al., 2022). By analyzing spatial sequences based on their syntactic structure, Wang et al. (2019) showed that activation in the bilateral IFG was modulated by the structural complexity of the sequences. This may relate to the human ability to spontaneously learn structural representations of visuospatial and auditory sequences by compressing these stimuli into language-like recursive codes (Al Roumi et al., 2023, Amalric et al., 2017, Planton et al., 2021). These findings highlight the human ability to learn domain-general sequential structures with a hierarchical organization.

### 6.2 Structure-based prediction

It is important to note that structural and predictive processing are not mutually exclusive. The structure of a given sequential signal may influence the probability distribution of upcoming inputs. Studies grounded in information theory have attempted to integrate structural and predictive frameworks by modeling input distributions using structural information. For example, Hale et al. (2018) modeled EEG responses during a story listening task using the surprisal metric derived from Recurrent Neural Network Grammars (Dyer et al., 2016). Subsequent studies employed LLMs to predict brain activity associated with the surprisal metric (Brennan and Hale, 2019, Goldstein et al., 2022, Heilbron et al., 2022, Schmitt et al., 2021, Tuckute et al., 2024). Notably, Brennan and Hale (2019) reported that models capturing the hierarchical structure of sentence stimuli achieved higher predictive performance. Goldstein et al. (2022) also demonstrated that an LLM-based model incorporating contextual information predicted brain activity more accurately than a model using only local semantic embeddings, suggesting that predictive processing in language likely depends on the hierarchical structure of sequential signals. While the structural and predictive frameworks differ in that they focus on the global relationships among elements of sequential signals and the local dynamics between them, respectively, integrating these two perspectives may provide a unified account of information processing across multiple temporal scales.

Meanwhile, a substantial portion of studies within the predictive framework is grounded in Markov models. For example, the free energy principle, a prominent theory within the predictive framework, assumes a Markov system as the underlying signal generation model (Friston et al., 2017); see Parr et al. (2025) for a recent proposal to revise this assumption. Statistical learning in both language and music has also been modeled using Markov models (Daikoku, 2018). However, the structural properties of sequential signals that involve long-distance dependencies cannot be explained by Markov models, as these models assume that the next element is determined on the basis of a fixed-length context. In contrast, long-distance dependencies involve elements that may be separated by an arbitrary number of embedded constituents of variable length.

This seemingly contradictory situation may arise from the coexistence of a statistical learning system based on a Markovian process and a compressive coding system that relies on the hierarchical structure of learned signal sequences. Indeed, studies have shown that statistical learning may contribute to the formation of hierarchical chunking (Daikoku et al., 2021, Wiggins, 2020). During statistical learning, neural oscillations initially synchronize with the frequencies of discrete tones or syllables (Ringer et al., 2025, Smalle et al., 2022). As learning progresses, these oscillations gradually synchronize with higher-order statistical groupings, such as words or phrases, reflecting the hierarchical organization of the acquired patterns. Furthermore, using the Hierarchical Bayesian Statistical Learning (HBSL) model with nursery rhyme data from Japan, France, Germany, Korea, and the UK, Daikoku (2023) demonstrated that repeated statistical learning led to increases in both the number and depth of hierarchical chunking through musical statistical learning.

In contrast, when recognizing previously learned sequential signals, or even during the learning process, humans tend to impose recursive hierarchical structures on these signals and compress the information (Al Roumi et al., 2023, Amalric et al., 2017, Planton et al., 2021). This aligns with the *dendrophilia* (“tree-loving”) hypothesis (Fitch, 2014), which posits a human-specific capacity and tendency to derive hierarchical tree structures from linear sequences. Indeed, cultural transmission experiments have demonstrated the spontaneous emergence of language-like structures from artificial symbol sequences (Kirby et al., 2015, Kirby et al., 2008, Saldana et al., 2019). This human capacity for structure generation, when combined with statistical learning abilities, may enable structure-based predictive processing across various cognitive domains.

### 6.3 Limitations and future directions

Although we believe that a series of neuroimaging studies partially supports our hypothesis of shared sequential signal processing across language, music, and mathematics, notable variations in brain activity patterns suggest possible inconsistencies among these cognitive domains. For example, while language stimuli can be presented in both visual and auditory modalities, mathematical stimuli are typically presented visually, whereas musical stimuli are predominantly auditory. Several previous studies have employed quantity perception and mathematical tasks in the auditory modality (Amalric and Dehaene, 2016, Eger et al., 2003, Piazza et al., 2006), suggesting that extending the sequential signal processing paradigm to auditory mathematics is feasible. In contrast, visual testing of music perception is more challenging, as understanding musical structure solely through sheet music generally requires extensive training (but see Horisawa et al. 2025 for a neuroimaging study on sheet music reading). Future research involving mathematical or musical experts may help uncover commonalities among these cognitive domains.

These cognitive domains also differ in terms of cultural universality. Despite their great diversity, language and music are observed across all human cultures worldwide (Evans and Levinson, 2009, Jacoby et al., 2024, Mehr et al., 2019). In contrast, complex calculations and symbolic algebra are relatively recent cultural inventions. With the exception of a limited number expression (Pica et al., 2004), symbolic mathematics is not universally observed across human societies. However, as Dehaene et al., 2025, Dehaene et al., 2022 argue, if the recognition of geometric shapes engages the same neural mechanisms as those used for symbolic mathematics, then the processing mechanisms of sequential signals may be universally involved in mathematics. This is supported by the presence of geometric shapes across human societies, even in cave paintings that predate the invention of symbolic mathematics (Dehaene et al., 2022).

Some may argue that mathematical expressions cannot be properly treated as sequential signals in the same way as music and language. We acknowledge that mathematical expressions, such as “2 × 4 + 8”, represent mathematical identities rather than temporal or procedural sequences. Nevertheless, previous studies have shown that people often process such expressions sequentially from left to right (Jansen et al., 2007, Schneider et al., 2012). We do not claim that all aspects of mathematics can be explained in terms of structure and prediction. Rather, we suggest that the processing of mathematical expressions may share some properties with language and music. Furthermore, although our examples primarily focus on arithmetic, this is because most research in the field of mathematical cognition typically uses arithmetic tasks. However, we have demonstrated that algebraic equations can be analyzed with the same linguistic tools as arithmetic expressions (Matsumoto and Nakai, 2023). Brain activity in the left IFG has been observed in response to algebraic expressions without numerical content (Friedrich and Friederici, 2009). Nonetheless, only a limited body of neuroimaging research has so far examined abstract mathematical expressions. This is partly due to the difficulties in designing such tasks and recruiting participants with sufficient mathematical proficiency. Overcoming this limitation will enable a more comprehensive understanding of the neural mechanisms underlying the processing of mathematical expressions.

To clarify whether language, music, and mathematics share similar neural processing mechanisms, cross-domain modeling will be a valuable approach in future research (Peelen and Downing, 2023). In cross-domain modeling, a model trained on one domain (e.g., language) is applied to another (e.g., mathematics) to predict brain activity or decode stimuli. Successful model generalization suggests that the domains share common neural representations. For example, using cross-modal encoding models, Nakai et al. (2021) revealed that both text and speech comprehension rely on common semantic representations in the perisylvian regions. Nakai et al. (2023) reported cross-format decoding between symbolic and nonsymbolic numbers in 5-year-old children, indicating that similar representations are shared across different number formats. In contrast to these cross-modal and cross-format studies, a major challenge in cross-domain analysis is how to extract common features from stimuli across different domains. This issue may be addressed using LLMs. Recent studies have shown that LLMs are capable of processing not only language stimuli but also mathematical problems to a certain extent (Yang et al., 2024, Zong and Krishnamachari, 2023). By embedding stimuli from multiple domains into a shared latent space via such LLMs, it becomes possible to test the generalization of computational models across sequential signals in different cognitive domains. However, it is also necessary to note certain reservations regarding the validity of LLMs as models of sequential signal processing. Artificial neural networks are often described as “black boxes” (Ras et al., 2022) and are trained on vastly larger datasets than those accessible to humans (Warstadt et al., 2025). Moreover, they often make errors in simple numerical reasoning tasks (Fujisawa and Kanai, 2022). Therefore, even if an LLM achieves higher predictive accuracy for brain activity compared to more interpretable models (such as Markov models), caution is warranted in interpreting whether the underlying algorithms of the LLM truly correspond to the neural mechanisms.

An important remaining challenge is to uncover detailed cortical implementations underlying the neuroimaging reports summarized in this review, as well as their connections to theoretical accounts of neural information processing. According to the standard predictive coding framework, deep cortical layers provide top–down predictions, while superficial layers carry bottom–up prediction errors (Bastos et al., 2012, Friston and Kiebel, 2009, Keller and Mrsic-Flogel, 2018, Spratling, 2017). Recent laminar fMRI techniques, which leverage ultra–high-field MRI to resolve layer-specific brain activity, could be a promising way to test this framework (Stephan et al., 2019). For instance, Thomas et al. (2024) demonstrated that expected Gabor orientations were equally decodable across V1 layers, whereas unexpected orientations were selectively decodable in superficial layers, supporting the distinct layer representations of predictions and prediction errors. Although it remains unclear whether a similar layer-wise functional organization holds for sequential signals, such laminar fMRI techniques, combined with modeling approaches to the electrophysiological responses (Nour Eddine et al., 2024), may lead to a more mechanistic understanding of sequential signal prediction in the human brain.

## 7 Conclusions

Human sequential signals, including language, music, and mathematics, share common structural and predictive mechanisms. Neuroimaging research has identified potential shared brain mechanisms across domains, while computational modeling and information-theoretic analyses have facilitated the integration of structural and predictive frameworks. The human capacity to generate hierarchical structures, combined with statistical learning, may support structure-based prediction. Cross-domain analyses will help resolve remaining inconsistencies across domains and further elucidate the neural mechanisms underlying sequential signal processing.

## CRediT authorship contribution statement

**Yohei Oseki:** Writing - review & editing, Conceptualization. **Tatsuya Daikoku:** Writing – original draft, Conceptualization. **Tomoya Nakai:** Writing – review & editing, Writing – original draft, Visualization, Project administration, Funding acquisition, Conceptualization.

## Declaration of Competing Interest

The authors declare no competing interests.
